# Impact of Continuing Medical Education (CME)-Based Sensitization on Problematic Smartphone Use (PSU) Among Medical Students: A Longitudinal Study

**DOI:** 10.7759/cureus.83695

**Published:** 2025-05-07

**Authors:** Anjum Datta, Sunaina Soni, Kiran Singh, Anusha Singh

**Affiliations:** 1 Physiology, Subharti Medical College, Swami Vivekanand Subharti University (SVSU), Meerut, IND

**Keywords:** deaddiction, roc curve, screen time, sdg-3, smartphone addiction scale

## Abstract

Background: Smartphones are being used both for leisure and learning, with young people being the most common users. Problematic smartphone use (PSU) negatively affects mental state, sleep, cognition, and academic achievement in college students. Therefore, in the present study, we assessed PSU in medical students before and after conducting a continuing medical education (CME) on “Achieving Sustainable Development Goal 3 (SDG-3): Deaddiction of drugs, alcohol, tobacco, and Smartphones”.

Materials and methods: Eighty medical students (aged 18-20 years) participated in the study. A CME was conducted at a tertiary care medical school in India in collaboration with the Heal Foundation and Asian Coalition for Health Empowerment, aligning with SDG-3 and the Doctor Against Addiction (DaAD) campaign. Smartphone addiction scale-short version (SAS-SV) was administered, and the screen time was recorded using “Your Hour,” a phone addiction tracker, and “Screen Time,” an iPhone application, before and after 15 days of CME. The data were analyzed using Statistical Package for Social Sciences (SPSS) (IBM Corp., Armonk, New York).

Results: The mean age of students was 18.9(0.6) years. A paired t-test was used to compare the mean SAS-SV score and screen time before and after CME. There was a significant reduction in the screen time t (78) = 9.686, p<0.001, after CME. However, the SAS-SV score was not significantly reduced. A Cronbach’s alpha correction coefficient of 0.872 for SAS-SV suggests good internal consistency. The receiver operating characteristic (ROC) analysis showed that the area under the curve (AUC) was 0.815 (95% CI, 0.750-0.880) with a cut-off SAS-SV score of 29.50 (sensitivity: 0.902; specificity: 0.620).

Conclusion: The results of the present study suggest that CME on de-addiction served as an effective intervention for sensitization of students, as evident from the reduced screen time. Further, SAS-SV could serve as a valid and reliable tool to assess PSU. Therefore, the need of the hour is to create awareness about PSU among the youth and make them aware of self-assessment tools such as phone apps to track screen time.

## Introduction

Smartphones have become an integral part of our lives as they are used both for leisure and learning. About 83.72% of the world’s population use smartphones, with young people being the most common users, as per Statista report, 2022 [1]. According to a WHO factsheet, people are more likely to have access to a mobile phone than to clean water [2].

Smartphone use has its pros and cons. People use their smartphones while walking, driving, sleeping, in lecture theaters, etc. The excessive use of smartphones to a level where they interfere with the daily lives of users is considered problematic smartphone use (PSU) [3]. An Indian study (2020) reported that 24.2% of the study population had smartphone addiction. Gender, age of exposure to smartphones, income, and residence in a city/village could serve as predictors of PSU [4].

Further, excessive use of smartphones has been associated with stress, anxiety, depression, sleep disturbances, aggressiveness, impulsivity, ocular manifestations, musculoskeletal disorders, reduced physical activity, obesity, social disturbances, and a decline in quality of life [5,6]. Recent literature has reported a negative effect on cognition and academic achievement in college students with smartphone addiction [7,8]. Therefore, the need of the hour is to create awareness about smartphone addiction among the youth and make them aware of self-assessment tools such as phone apps to track screen time.

Continuing medical education (CME) has been considered an effective learning method in the medical community for updating the knowledge and skills of students, residents, and even clinicians periodically [9,10]. Further, adding interactive activities to didactic lectures, as in CME, reinforces the teaching and improves the outcome of the CME [11].

Therefore, in the present study, a CME talk was organized to sensitize medical students about smartphone addiction and cognitive dysfunction, and the PSU was assessed. The screen time was measured objectively using a phone app and smartphone addiction scale-short version (SAS-SV) score; a subjective reporting method was used before and after 15 days of CME in order to investigate the impact of CME on their smartphone usage. Further, we hypothesized that both screen time and the SAS-SV score of medical students would reduce after attending CME.

## Materials and methods

Subjects

Eighty medical students aged between 18-20 years were recruited for this longitudinal study after obtaining their written informed consent. The study was conducted from June to July 2024 in the Department of Physiology at a tertiary care medical school in India. All participants had no history of significant neurological and medical illness or substance use disorders and were not on any type of medication. The study was conducted as per the ethical guidelines of the Declaration of Helsinki and was approved by the Institutional Ethics Committee (SMC/UECM/2024/832).

Continuing medical education (CME)

A one-day CME was conducted at a tertiary care medical school in India in collaboration with the Heal Foundation and the Asian Coalition for Health Empowerment, which aligned with Sustainable Development Goal (SDG-3) and the Doctor Against Addiction (DaAD) campaign. The topic of the CME was “Achieving Sustainable Development Goal 3 (SDG-3): De-addiction of drugs, alcohol, tobacco, and smartphones”. There were four sessions in CME, one on each type of addiction, i.e., drugs, alcohol, tobacco, and smartphones. The talk on “Smartphone addiction and cognitive dysfunction” was converted into an interactive session, and the students were educated on the pros and cons of excessive smartphone use.

Assessment of screen time and problematic smartphone use (PSU)

Students were told to install a phone application “Your Hour,” which is a phone addiction tracker (Mind-e-fy Solutions, Madhya Pradesh, India) for Android users, and iPhone users were instructed to use “screen time,” a built-in application on iPhone for iOS to objectively measure screen time. 

Smartphone addiction scale-short version (SAS-SV), a valid questionnaire to elaborate on the characteristics of smartphone usage, was administered. The scale includes 10 questions. For each item, participants responded on a Likert scale ranging from 1 (strongly disagree) to 6 (strongly agree) (see Appendix, Table 4). The sum of all items gives an overall score of 60, with a higher score indicating PSU [12]. Screen time and SAS-SV score were recorded before and after 15 days of CME. A Google Form (Google, Mountain View, California) was created to collect the data from the students before and after 15 days of the CME.

Statistical analysis

Statistical analysis was carried out using Statistical Package for Social Sciences (SPSS) (IBM Corp., Armonk, New York). Data were examined for the assumptions of normality using the Shapiro‐Wilk test statistic and homogeneity of variances using Levene's test. The mean screen time and SAS-SV score were calculated for before and after CME data, and the means were compared for the two time points for statistical significance using a paired-sample t-test. A p-value of <0.05 was considered to report statistically significant findings.

The reliability of the questionnaire was assessed using internal consistency, which is an index indicating the homogeneity of the items of an instrument. In this method, Cronbach’s alpha (α) coefficients > 0.7 represent acceptable reliability [13].

The receiver operating characteristic (ROC) curve was analyzed to examine the diagnostic ability of the SAS-SV for predicting PSU. The area under the curve (AUC) of the ROC is a measurement of the diagnostic ability of the SAS-SV score in order to correctly classify a specified outcome of PSU. A value of 1.0 is considered a perfect test, whereas a value of 0.5 or smaller is considered similar to a random guess or flipping a coin based on its inconsistency. The guidelines for interpreting the AUC values were as follows: AUC of 0.7-0.8 is acceptable, AUC of 0.8-0.9 is excellent, and AUC of 0.9 or greater is outstanding [14]. The value corresponding to the nearest point of the ROC curve to the top left-hand corner was chosen as the optimal cut-off for predicting PSU, which maximizes both sensitivity and specificity.

**Figure 1:**
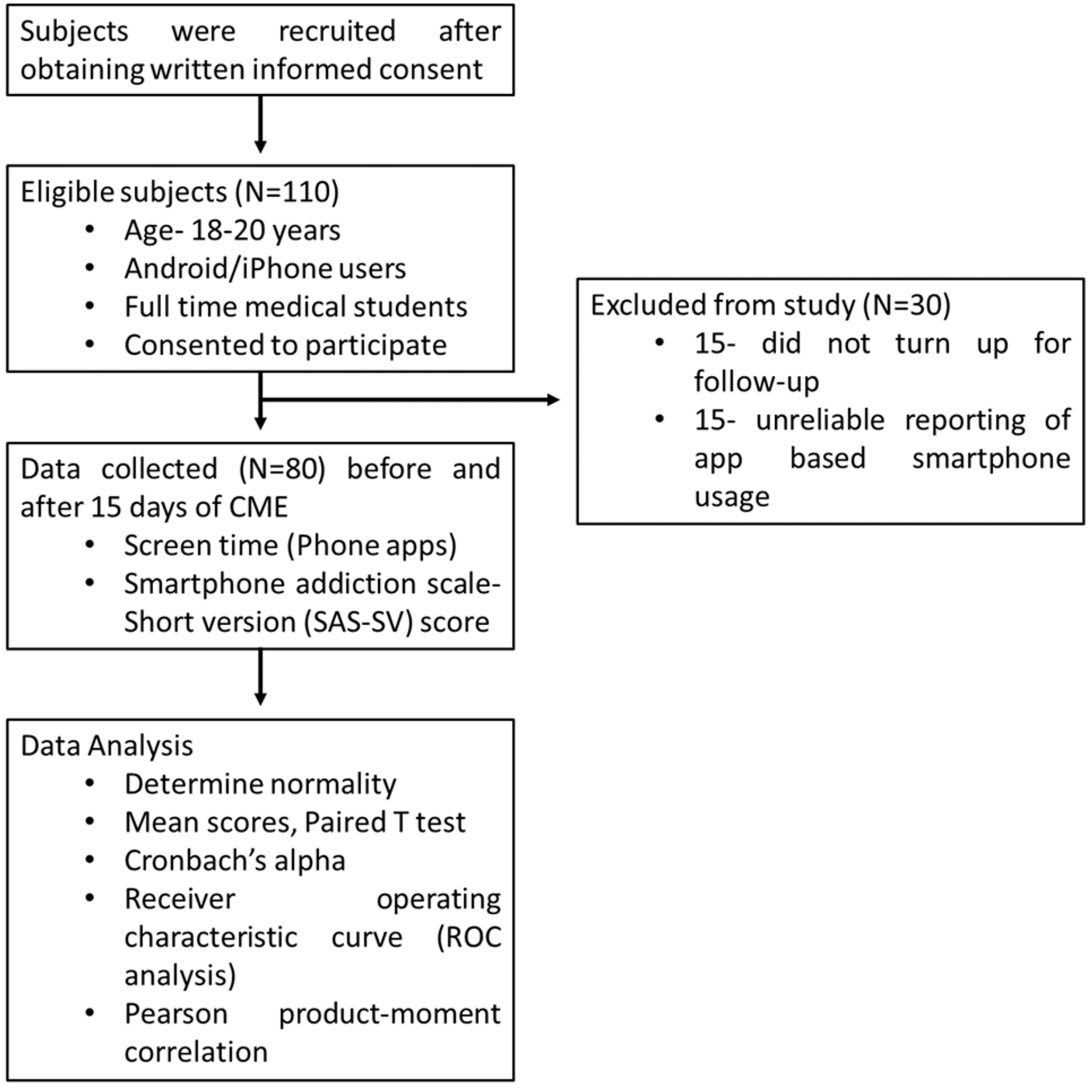
A summary of the workflow of the study. CME: continuing medical education.

Pearson product-moment correlation was conducted between screen time and SAS-SV score to determine the relationship between the two variables (Figure 1).

## Results

The mean age of students was 18.9(0.6) years. Forty-five female and thirty-five male students participated in the study. The data were normally distributed. There was a significant reduction in the screen time t (78) = 9.686, p<0.001, after CME with a large effect size, Cohen’s d = 0.9. However, the smartphone addiction scale-short version (SAS-SV) score was not significantly reduced, t (78) = 1.875, p = 0.065 (Table 1).

**Table 1:** Paired samples t-test. SAS-SV: smartphone addiction scale-short version.

	Mean (std. error)	95% Confidence interval of the difference		t value	p value
		Lower	Upper		
Screen time (before-after) in minutes	154.772 (15.97)	122.959	186.585	9.686	<0.001
SAS-SV score (before-after)	1.405 (0.749)	–0.087	2.897	1.875	0.065

A Cronbach’s alpha correction coefficient of 0.872 was obtained for SAS-SV, which suggests good internal consistency. The corrected item-total correlation coefficients ranged from 0.556 to 0.671 and were within the acceptable range (Table 2).

**Table 2:** Reliability statistics of problematic smartphone use (PSU)-Cronbach’s alpha.

Question no.	Scale mean if the item deleted	Scale variance if the item deleted	Corrected item-total correlation	Squared multiple correlation	Cronbach's alpha if the item deleted
1	27.85	79.475	0.556	0.490	0.863
2	28.45	76.797	0.632	0.482	0.857
3	28.54	78.988	0.512	0.332	0.867
4	28.78	79.125	0.543	0.425	0.864
5	29.04	78.221	0.619	0.528	0.858
6	29.09	78.876	0.619	0.531	0.858
7	29.23	78.830	0.625	0.482	0.858
8	28.56	80.134	0.495	0.277	0.868
9	27.63	77.546	0.666	0.497	0.855
10	28.81	76.817	0.671	0.544	0.854

**Figure 2:**
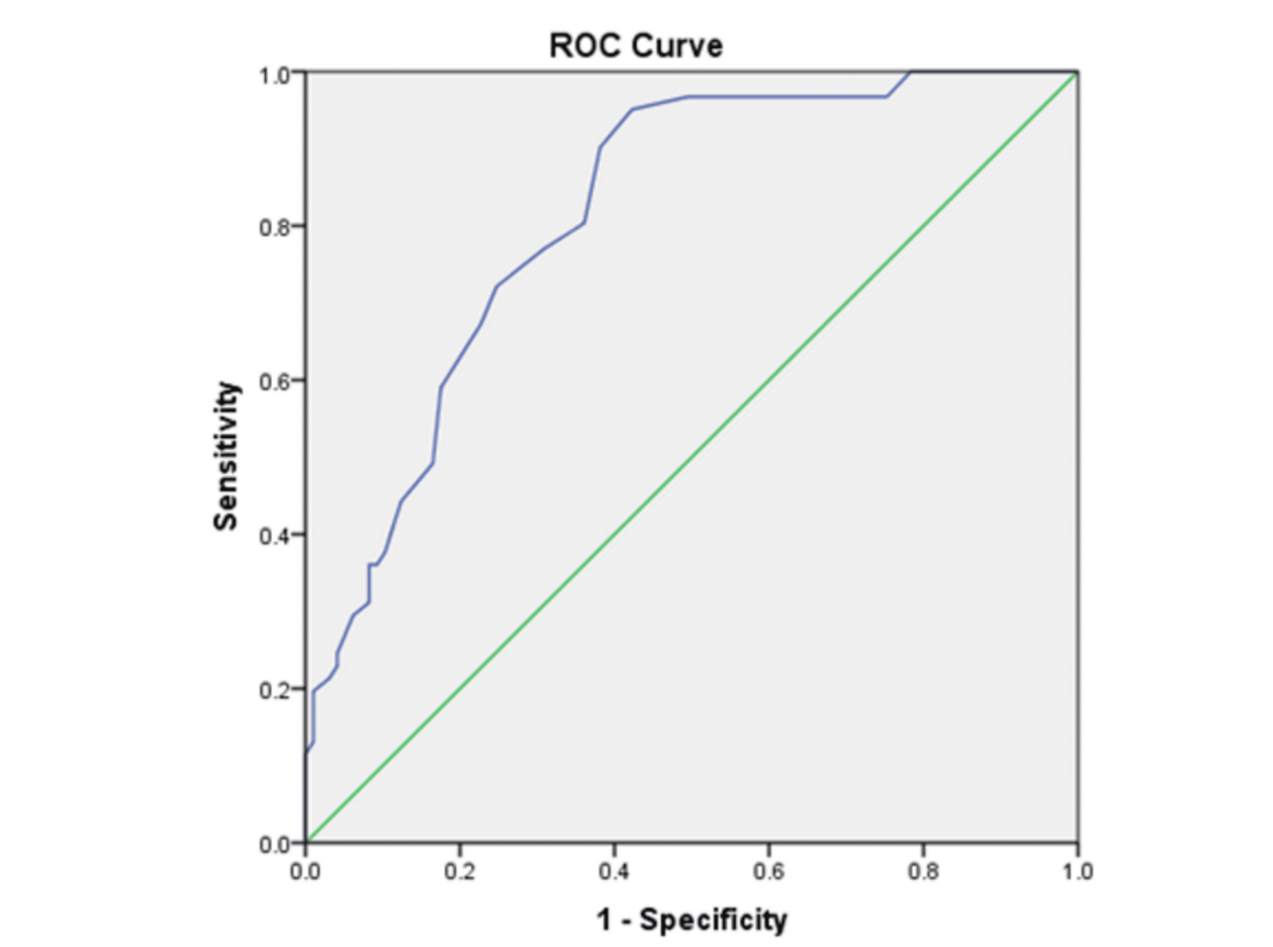
Receiver operating characteristic (ROC) curve for SAS-SV score. SAS-SV: smartphone addiction scale-short version.

The receiver operating characteristic (ROC) curve was analyzed for the SAS-SV score. The area under the curve (AUC) was 0.815 (95% CI, 0.750-0.880) with a cut-off SAS-SV score of 29.50, for which sensitivity was 0.902 and specificity was 0.620 (Table 3, Figure 2). According to our findings, 62.5% of participants were found to have higher than 29.50 scores on SAS-SV.

**Table 3:** Sensitivity, specificity, and cut-off point according to the receiver operating characteristic (ROC) curve.

Cut-off point	Sensitivity	1-Specificity
21.50	1.000	0.784
22.50	0.967	0.753
23.50	0.967	0.711
24.50	0.967	0.649
25.50	0.967	0.639
26.50	0.967	0.557
27.50	0.967	0.495
28.50	0.951	0.423
29.50	0.902	0.381
30.50	0.803	0.361
31.50	0.770	0.309
32.50	0.721	0.247

A Pearson product-moment correlation was run to determine the relationship between screen time and SAS-SV score. There was a weak positive correlation found with Pearson’s correlation coefficient r = 0.25, which was statistically significant at p = 0.002. 

## Discussion

The present study was conducted to investigate the effect of CME-based sensitization on problematic smartphone use (PSU) in medical students before and after 15 days of CME. The main findings of the study suggest that there was a significant reduction of screen time with a large effect size; however, the SAS-SV score reduction was not significant after the CME. The cut-off score for SAS-SV was found to be 29.5 for our study sample using ROC analysis, and about 62.5% of participants scored higher than 29.5 on SAS-SV.

Smartphone use has its pros and cons. It is beneficial for accessing educational resources, which often leads to excessive and non-academic usage, including social media, messaging, and entertainment applications. Students end up in compulsive checking, anxiety when away from their phones, and difficulty in regulating their screen time.

Screen time

Previous studies have suggested that longer screen time was associated with reduced physical activity, more sedentary behavior, poor mood, sleep quality [15,16], and academic stress score [17]. A recent systematic review has reported that excessive screen time can be associated with attention problems in children [18]. A strong correlation was suggested between increased screen time and the level of problematic smartphone use in students of medicine [19] as opposed to a weak positive correlation derived from the findings of our study. Further, a nudge-based intervention was used to reduce PSU, and after six weeks, it was found to be effective in lowering the screen time, reducing PSU, and improving sleep quality [20], similar to our study findings. Therefore, the present study results suggest that CME-based sensitization may serve as an effective intervention to reduce screen time in students.

SAS-SV score

Our study findings suggested a cut-off of 29.5 for the smartphone addiction scale-short version (SAS-SV) score to identify problematic smartphone use (PSU), based on which more than half of the study participants could be categorized as addicted to smartphones. On the contrary, a higher cut-off value for SAS-SV was suggested by Kwon et al., i.e., >31 for males and >33 for females to predict smartphone addiction [12]. According to our study sample’s cut-off value, 62.5% of the medical students were addicted to smartphones as opposed to previous literature, which suggests 52% [21] and 44% [5] in India. Further, a higher score on SAS-SV was found to be associated with decreased physical activity, increased BMI, poor sleep quality [22], and poor attention control [23] in young adults.

Limitations

In the present study, a self-reported smartphone addiction questionnaire was used, and it is known that people always underestimate their actual smartphone usage, giving rise to subjective bias. In the future, the same study can be conducted using a robust study design, i.e., randomized controlled trial (RCT), which will provide reliable and valid findings and reduce recruitment bias. Further, the gender variation with regard to PSU and the effect over a long follow-up period could be studied.

## Conclusions

Smartphones have become an integral part of students’ daily life, facilitating work, education, or entertainment. However, they have to be cautious in smartphone use, as the results of the present study suggested that a significant number of students are facing problematic smartphone use (PSU). It is important not only to utilize the advantages of the smartphone but also to reduce the negative consequences. Therefore, the need of the hour is to create awareness about PSU in the youth, encourage digital wellness and make them aware of valid and reliable self-assessment tools such as phone apps to track screen time and scales like SAS-SV.

## Appendices

**Table 4:** English version of smartphone addiction scale-short version. Reprinted with permission from Kwon et al. [12]. This is an open-access article distributed under the terms of the Creative Commons Attribution License, which permits unrestricted use, distribution, and reproduction in any medium, provided the original author and source are credited.

S. no.	Items	Strongly disagree	Disagree	Weakly disagree	Weakly agree	Agree	Strongly agree
1	Missing planned work due to smartphone use	1	2	3	4	5	6
2	Having a hard time concentrating in class, while doing assignments, or while working due to smartphone use	1	2	3	4	5	6
3	Feeling pain in the wrists or at the back of the neck while using a smartphone	1	2	3	4	5	6
4	Won’t be able to stand not having a smartphone	1	2	3	4	5	6
5	Feeling impatient and fretful when I am not holding my smartphone	1	2	3	4	5	6
6	Having my smartphone in my mind even when I am not using it	1	2	3	4	5	6
7	I will never give up using my smartphone even when my daily life is already greatly affected by it	1	2	3	4	5	6
8	Constantly checking my smartphone so as not to miss conversations between other people on Twitter or Facebook	1	2	3	4	5	6
9	Using my smartphone longer than I had intended	1	2	3	4	5	6
10	The people around me tell me that I use my smartphone too much	1	2	3	4	5	6
